# Supplementary material for: The IUCN Red List and newspaper coverage of threatened freshwater eel species in Japan: A variable but limited influence
Source: PLoS One. 2025 Oct 29;20(10):e0331511. doi: 10.1371/journal.pone.0331511 (PMC12571305; doi:10.1371/journal.pone.0331511)
Supplement: Attachment — Submitted filename: Expert Review Report PONE-D-25-32104_reviewer.pdf [file pone.0331511.s002.pdf]

Expert Review Report

Article with comments: PONE-D-25-32104_reviewer PDF

Preface

The study presented in this article is both complex and compelling, characterized by an investigative approach through the analysis of written sources (newspapers and other media). It addresses a major scientific and societal issue: biodiversity conservation amid increasing extinction threats. The IUCN Red List is recognized worldwide as the standard for assessing species extinction risk, playing a key role in raising public awareness and guiding conservation policies. However, the actual impact of this tool on societal and media perceptions remains under-documented, especially over the long term and within specific cultural contexts.

This article explores this issue through an original investigation of Japanese media coverage of freshwater eels, a species emblematic ecologically, culturally, and economically. The longitudinal approach (1992–2021), combining quantitative and qualitative analyses of print media, is complex but offers valuable insights into how conservation lists influence public awareness within a multifaceted and evolving media environment.

Scientific Context and Summary

- Clear and rigorous presentation of the IUCN Red List as the global standard for extinction risk assessment.
- Relevant emphasis on the Red List's role in public awareness, a crucial component of conservation efforts.
- Well-justified choice of freshwater eels as a case study, with detailed ecological, cultural, and economic context, particularly in Japan.
- Extended 30-year temporal analysis enabling observation of trends before and after species listing.
- Use of advanced text mining tools to analyze media coverage.
- Rigorous methodology combining data collection from major Japanese newspapers, manual filtering, robust statistical analyses (Rosner's test, Generalized Additive Models), and complementary qualitative analysis.

- Clear identification of major media events (2008 commercial scandal, 2014 Red List impact) with coherent thematic classification.

Comments on the Abstract (Lines 26–39)

- Soften the phrase "***essential tool***" to "***important tool with variable impact***" (**L26–27**).
- Specify the duration and nature of the described "***momentary***" media impact (**L30–34; L36–37**).
- Clarify the limited coverage of non-native species to avoid ambiguity (**L32–34**).
- Indicate whether the dominance of "***food***" and "***commerce***" themes changes over time (**L34–36**).
- Better align the recommendations with the study's findings (**L37–39**).

1. Introduction

Reservations and Contradictions

- **L55-59:** Uncertainty about the actual influence of the Red List on societal attitudes and political decisions, tempering its attributed importance.
- **L63-66:** Potential bias from using Internet search trends as proxies for public awareness.
- **L67-70:** Limitations of interview surveys regarding sample size and geographic scope.
- **L70–75; 83–94:** Exclusive focus on Japanese print media restricts representativeness and geographic scope.
- Lack of analysis on external factors potentially affecting media coverage (e.g., political campaigns, health crises).

Suggestions

- Incorporate digital and social media to improve representativeness.
- Expand geographic scope to include other cultural contexts.
- Account for external factors influencing media coverage.

2. Materials and Methods

Reservations and Contradictions

- **L185–189:** Keywords used for thematic classification are mainly food-related, potentially biasing conservation theme representation.

- **L204–206:** Keyword selection described as ***"arbitrary,"*** introducing subjective bias.
- **L222–225:** Species-specific analysis limited to four Anguilla species out of sixteen, restricting scope.
- **L290–300:** Qualitative analysis sample size (40 articles per outlier year) may be insufficient for comprehensive representation.
- As noted in a comment on the introduction, no explicit consideration was given to external factors influencing media attention (e.g., political events, health crises).
- Lack of detail on validation of statistical assumptions (linearity, residual independence).

Suggestions

- Broaden keyword lexicon to include conservation and protection terms.
- I am not a specialist in language statistics or text processing, but according to current documentation, the use of automated methods (e.g., TF-IDF, machine learning) helps reduce keyword selection bias.
- Extend analysis to all IUCN-evaluated Anguilla species.
- Increase qualitative sample size and representativeness.
- Integrate external variables into statistical models.
- Verify and document statistical assumptions rigorously.

3. Results and Discussion

Reservations and Contradictions

- **L350–365:** The major media peak in 2008 is primarily linked to a commercial scandal (mislabeling), possibly obscuring the direct effect of the Red List on awareness.
- **L332–335; 406–409:** The ***"Food"*** category dominates coverage, potentially overshadowing conservation issues.
- **L353–356:** Absence of outliers for the ***"Aquaculture"*** category suggests insufficient media attention to this critical aspect.

- **L371–381:** Insufficient discussion of other external factors (regulations, health crises) that may have influenced media coverage.
- **L412–414:** Very high explanatory power of the model (adjusted $R^2 = 0.996$) may indicate overfitting given the limited number of explanatory variables.

Suggestions

- Clearly distinguish media impacts of commercial scandals from conservation assessments.
- Promote more balanced media coverage including aquaculture and conservation topics.
- Incorporate external variables to better isolate the Red List's effect.
- Test model robustness and alternative models to avoid overfitting.

Conclusion

Despite the complexity inherent in multifactorial and longitudinal media analyses, **this study makes a valuable contribution to understanding the impact of conservation lists on public awareness**. Identified contradictions and limitations are primarily methodological and editorial, addressable through targeted revisions and clarifications.

Accordingly, **I recommend acceptance with minor revisions** to enhance clarity, rigor, and scope without compromising scientific validity. **This work merits publication as it opens promising avenues for future research and conservation communication.**

DM (Bsi in the comments)
