# Supplementary material for: The IUCN Red List and newspaper coverage of threatened freshwater eel species in Japan: A variable but limited influence
Source: PLoS One. 2025 Oct 29;20(10):e0331511. doi: 10.1371/journal.pone.0331511 (PMC12571305; doi:10.1371/journal.pone.0331511)
Supplement: Attachment — Submitted filename: Responses_to_reviewers.docx [file pone.0331511.s004.docx]

Thank you very much for your time and effort in reviewing our manuscript entitled "The IUCN Red List and newspaper coverage of threatened freshwater eel species in Japan: a variable but limited influence." We are sincerely grateful to the editor and reviewers for their thoughtful, constructive, and detailed comments. Your insights have significantly improved the clarity, rigour, and overall quality of our manuscript. We deeply appreciate the careful attention you have given to our work and the opportunity to revise and strengthen the paper in response to your suggestions. Please see the table below for our responses to the individual comments.

| Reviewer #1 | |
| --- | --- |
| This is a good paper and I recommend its publication with some suggestions and minor revision. I appreciate that the authors are looking at eel conservation drivers through popular media (newspapers). They provide a strong narrative through out the paper, explaining or hypothesizing about how article trends are reflected by conservation and fishery phenomenon. | Thank you very much for your kind and encouraging comments. We truly appreciate your positive evaluation of our study and your helpful suggestions, which have been valuable in improving the manuscript. |
| Line 34: You abstract mentions “food and trade dominated media coverage”. You touch on this in the Discussion, but do you think that this points the way to greater public awareness? Should more articles and events be centered around food, as a tool for ultimate conservations since it demonstrates a particular value to eels? | A recent study analysing newspaper articles on eels published in Japan over the past two years has suggested that continued public interest in eel consumption may contribute to conservation efforts. We have now cited this paper in the Discussion and incorporated a brief reflection on the potential role of food-related discourse in supporting eel conservation. Please see lines 654-659.  Our ongoing research, which combines focus group interviews and an online survey to investigate willingness to pay (WTP) for the sustainable use of eels, also shows a tendency for those who consume eels to exhibit higher WTP compared to those who do not. This study can be positioned as a follow-up to some of the findings presented in this paper, and your suggestion is therefore highly meaningful. We sincerely appreciate it. |
| Line 94: What is “production” here? Aquaculture within Japan, or harvested from Japan though raised in foreign aquaculture? All aquaculture eels? | To make the meaning clearer, I have revised the sentence as follows. Please see lines 92-95.  “According to FAO data, Japan accounted for approximately 70% of the global production of Anguilla species (combined capture fisheries and aquaculture) in 2000 and 2001.” |
| Line 101: Was the topic of eels most frequent among any fish species, of most frequent of any topic, fish-related or otherwise? | Eels ranked first among all fish species. I added 'among any fish species' to clarify this. Please see lines 98-102. |
| Line 121: Was this physical, digital, or both (I assume both)? | The source paper simply uses the term "circulation", so it is unclear whether it refers to print, digital, or both. Therefore, we aligned our wording with the original and avoided specifying whether it is print or digital. Please see lines 120-122. |
| Lines 136-165: This section is not bad here, but could be moved to the Introduction adjacent to the material in lines 76-87. | We discussed among the co-authors whether to include a detailed explanation about eels in the Introduction or in the Materials and Methods section. As the explanation was rather lengthy and would interrupt the logical flow from background to objectives of our study, we decided to place it in the Materials and Methods section instead. Since you also found it is not bad to include this information there, we have decided to keep it as is without further revision. |
| Line 168: Table 1. Just to check, for row 2012, column “Global”, are you talking about US inclusion under CITES, or the US Endangered Species Act? | We are talking about US inclusion under CITES Appendix II. Plese refer to a link below. <https://safmc.net/documents/protected-resources-committee-usfws-final-eel-and-loggerhead-briefing-statement-june-13-2013-doc-pdf/> |
| Line 214: Table 2. Is there any additional explanation as to why many categories have multiple key words, while “Mislabeling of origin” and “Fishery” only have on each? | The reason is that these two categories have corresponding Japanese terms with a one-to-one match to their category names, and articles in these categories almost always include those keywords. This explanation has been added to the description of Table 2. Moreover, we confirmed that adding the keywords did not change the number of articles in each category. Please see lines 215-221. |
| Line 225: Add “eel” after “shortfin”. | Thank you for pointing out the spelling error. |
| Line 276: Do you mean “each” as in all of the four eel species, or should “each” be replaced with “any”? | No, here we intended to refer to the number of articles in which each eel species appears. To make the meaning clearer, we have revised the sentence as follows. Please see lines 278-281.  For the species-specific analysis, a linear multiple regression model was applied, with the yearly total number of articles as the response variable and the number of articles in which each of the four eel species was mentioned in each year as explanatory variables. |
| Line 289: I keep getting hung up on the term “outlier”. When I see the charts of newspaper articles, these data points strike me as “peaks” or “high points” (as you do in Line 325). The term “outlier” feels like it has a context of being way outside norms, something that has to be “adjusted” for. You are technically correct with your way of using it, but I keep stumbling at it because its used so often. | Thank you for your comment. You are right that the term "outlier" may give the impression of data points that are inappropriate for analysis. Therefore, we have decided to use your suggested term "peak" instead. The wording was amended thoughout the manuscript.  An explanation was added in lines 266-267. |
| Line 407: I think this high count of article on “food” and “trade” could be examined a little more in the discussion. Future research or even conservation actions could focus on food and trade, and how demonstrating the value of eels as a food source, and writing about this, could reach many more Japanese people. | Please refer to our response for the comment on line 34. |
| Line 413: Does “these categories” refer to the food and trade categories? If so, state that. | “These categories” refer to six categories use in the GAM analysis as explanately variables. To clarify this, we insert ‘six’ between “these categories.” Please see lines 418-421. |
| Line 457: The main point might be just as strong if you gave one total percentage, not divided into separate species. | We reviewed 76 articles published in 2014 that mentioned the four eel species and noted that 41 of them (53.9%) included references to the IUCN. We also stated that, since the IUCN’s assessment results for eels were published on 12 June 2014, these articles were written more than five months after the release. Please see lines 461-463. |
| Line 532-536: Excellent points. | Thank you very much! We are also pleased to have made such a positive finding. |
| Line 561-563: Yes, but isn’t that the point, of even the definition, of “news”? | As you rightly pointed out, it may be somewhat self-evident, but since it also relates to the peak detection method used in this study, we have taken care to explain it thoroughly. |
|  |  |
| Reviewer #2 | |
| This manuscript stands out for its high methodological quality and its original contribution to the study of the societal impact of conservation lists, particularly within the Japanese context and over a span of three decades. The longitudinal approach, combining quantitative and qualitative analyses, allows for an in-depth documentation of the evolution of media coverage and public perception surrounding an emblematic species. | Thank you for your kind and insightful feedback. We truly appreciate your recognition of the originality and methodological rigour of our study, as well as your thoughtful remarks on its relevance to understanding long-term media trends in conservation within the Japanese context. |
| 1 : The manuscript is generally technically sound, and the data support the conclusions. The study employs a robust methodology combining quantitative and qualitative analyses over a long period, which contributes to the credibility of the results. However, some methodological limitations—such as potential biases in keyword selection and limited consideration of external influencing factors—should be acknowledged. These do not invalidate the findings but suggest that conclusions should be interpreted with appropriate caution. | Thank you for your insightful observation, which we fully acknowledge and value. While we have summarised the findings of this study at the end of the Discussion, we have now added the following note of caution regarding the interpretation of the results. Please see lines 689-692.  “Finally, it should be noted that these conclusions should be interpreted with appropriate caution, as certain methodological limitations remain—such as potential biases in keyword selection and the limited consideration of external influencing factors.” |
| 2 : The statistical analysis was conducted appropriately and rigorously. The methods used, such as the Rosner test and GAM models, are suitable and carefully applied. The methodological remarks are minor and aim to further enhance robustness without questioning the validity of the results. | Thank you very much for your thorough examination of the methodology used in this study. We have revised the manuscript accordingly, including responses to the comments provided by Reviewer 1. We sincerely hope that these revisions appropriately address the thoughtful feedback we received. |
| 3 : In response to PLOS Data policy, the authors indicated that the data used in this study come from archived databases (Asahi Shimbun Cross-Search, Maisaku Database, Nikkei Telecom 21, and Yomidasu Rekishikan) that require a paid subscription. They do not have the authority to make these primary data publicly available due to access restrictions and contractual conditions imposed by the database providers. Given these legitimate constraints, it is understandable that the underlying data cannot be made fully accessible without restrictions. However, in accordance with PLOS requirements, the authors are expected to provide all derived data, analyses, and results necessary to reproduce the study, as well as clear information on how to access the original databases through the official providers. This approach balances the imperatives of transparency and reproducibility with the limitations imposed by third-party access rights. | We have considered your comment and added the URL of the database to the Materials and Methods section. Although access to the database requires a subscription, readers with access will be able to replicate the subsequent analyses. Please see lines 174-179. |
| 4 : As a non-native English speaker, and having already informed you that I use translation tools, assisted writing software, and artificial intelligence to translate the text and draft my review report, I find myself in a position where it is difficult to confidently assess the linguistic quality of the manuscript in English. Although the text appears generally clear and understandable, I am not able to fully evaluate grammatical accuracy, stylistic fluency, or the presence of subtle ambiguities that might require thorough revision by a native speaker or a professional language editor. | Thank you for your suggestion. This manuscript was proofread by a native English-speaking researcher from the United States prior to submission. In addition, Reviewer 1, who appears to be a native English speaker, kindly pointed out several grammatical issues. With the support we received, we believe the quality of the English in the manuscript has been significantly improved. |
| Therefore, I recommend that, should the manuscript be accepted, a dedicated language review be considered to ensure that the text fully meets PLOS ONE’s standards for clarity, correctness, and unambiguity. This will help guarantee an optimal presentation of the work to an international readership. | Thank you very much. We would greatly appreciate it if the publisher's English editing further improves the language quality of our manuscript. |
|  |  |
| Expert Review Report PONE-D-25-32104_reviewer | |
| Soften the phrase “*essential tool*” _to “*important tool with variable impact*” _(L26–27). | We searched through the entire manuscript, but the phrase "essential tool" was not found anywhere in our text. |
| Specify the duration and nature of the described “*momentary*” _media impact (L30–34; L36–37). | We searched through the entire manuscript, but the word "momentary" was not found anywhere in our text. |
| Clarify the limited coverage of non-native species to avoid ambiguity (L32–34). | Since the section you pointed out is the abstract, we have kept the explanation concise. As the details are thoroughly explained in the main text, we prefer to leave this part unchanged. |
| Regarding Introduction, Materials and Methods, Results, and Discussion. | The suggestions regarding the sections from Materials and Methods onwards are considered not so much as revisions to improve the current manuscript, but rather as valuable recommendations to enhance future studies based on this research. We, the authors, will carefully review and consider these suggestions as we develop our next research plan. We sincerely appreciate your insightful and important input. |
